# Physiology

**Published:** 1824-06-01

**Authors:** 


### Quarterly Periscope OF PRACTICAL MEDICINE;

BEING THE

### Spirit of the Public Journals,

FOREIGN AND DOMESTIC.

### I.

### Physiology.

——————— latas a numine leges
Religiosa doce, mentesque cupidine veri
Allice.

1. *Therapeutical Physiology, or Medical Miracles.** Who has not heard of the wonder-working prayers of Prince Hohenloe? Are we to consider these cures as falsehoods, and the parties, active and passive, as impostors? We are not of that opinion. We believe that some—perhaps most—of the cures were actually performed, and that through the agency of mind on matter. We cannot be so uncharitable as to suppose that the patients have wilfully deceived the world, however they may be deceived themselves in respect to the interference of a supernatural power in their behalf. We dare not say so much for the Intercessor. And yet it is possible that Dr. Hohenloe may be so vain and superstitious as to believe he has the ear of the Almighty in Heaven, in the same way as subjects are supposed to have the ear of Majesty on Earth.

These miraculous cures have not excited much attention in this country. They served to fill columns in the newspapers, and make the vulgar stare; but the philosophic part of the community looked upon them as the mere workings of the imagination on the corporeal fabric, instances of which are every day presented to the attentive observer. In Ireland, however, where the odium theologicum is heightened by the odium politicum, these occurrences have become a new cause of dissention, and have been discussed with all the bitterness of religious and political animosity.

The plenitude of power, in the miracle line, so long claimed by the Church of Rome, has dwindled, in modern times, to a very low ebb indeed. Is this to be attributed to the increase of *faith* in mankind,

* An attempt to explain, on natural principles, the cures alledged to be miraculous, of Miss Lalor and Mrs. Stuart. By a physician.

rendering divine manifestations unnecessary? or is it to be placed to the account of a prudent reluctance on the part of the priesthood to appeal to *miracles* in so *enquiring* an age as the present? Be that as it may, the exploits of Prince Hohenloe have revived the drooping spirits of the Roman Catholic Clergy, whose bishops, in their pastoral letters, "*announce, with great joy, a splendid miracle which the Almighty had wrought even in our own days, and in the midst of ourselves.**" This "splendid miracle," which we expected could not be less than a conversion of the river Shannon into claret, at least, resolves itself into the simple fact that, a young lady, Miss Lalor, recovered the use of her tongue after the dreadful punishment of six years' silence! We have little doubt that Miss Lalor will make up for lost time, now that she has recovered the use of such an important organ.

The pastoral letter of Bishop Doyle was soon followed by another from the pen of Arch-bishop Murray, announcing the miraculous cure of a nun in the convent of Ranelagh, effected "*by the supernatural interference of the divine power, through the intercession of Prince Hohenloe.*" The *credibility* of this last miracle is attempted to be strengthened by certain medical testimonies. According to Dr. Mills, "the complaint (of the Ranelagh nun) was generally of an apoplectic tendency;"—while, by Dr. Cheyne, the lady is described "*as being an ailing person, having laboured under determination of blood to the head, and various* NERVOUS *affections of an* ANOMALOUS *character.*" From this statement, not very clear in itself, it must be perfectly clear to our readers that the Ranelagh nun was a most excellent subject for a miracle. We accordingly find her "a great sufferer" on the 31st of July; but, mirabile dictu! on the 4th of August, she "*assured her physicians that she was without complaint.*" Why, the passage of the Red Sea by Moses, or the supply of manna to the Israelites in the wilderness, was nothing compared to this! That the splendor of such a miracle should dazzle the optics of the Catholics is not to be wondered at; and accordingly they see nothing in the transaction but "the finger of God"†—a proof "that the Almighty God had thus visited his people, reanimating their faith, and reviving their hope."‡ Yet there is a party of reasoners in the Emerald Isle, who, scorning all reference to natural principles, freely admit the reality of the miracles, but discover in them, not "the finger of GOD," but of "the DEVIL;" "to whom (it seems) the exertions of the bible society have given a most serious alarm," and therefore his Satanic majesty is lending a hand to the Catholics, whose object, of course, is to keep people in the dark! We accordingly find a red hot SAINT from the Bible Association assuring his reverend antagonist, Bishop Doyle, "that he has *great pleasure* in the miracles, inasmuch as it is evidence that the church whence they proceed is the MOTHER OF HARLOTS!!"

As we can have no hope of reconciling these ultra-superstitious par-

* Bishop of Leighlin and Fernes' pastoral letter.

† Archbishop Murray's letter. ‡ Bishop Doyle's letter.

ties to one another, we shall leave them to debate on the celestial or infernal origin of the miracles, while we revert upon our former declaration, (which is also the creed of the able author of the pamphlet before us,) namely, that the cures were real—not through the intercession but the *influence* of the Prince Hohenloe—and that the patients were not impostors. It is melancholy, however, to think that, in the present enlightened æra, there should be numbers of people, among the learned bodies too, so superstitious or rather so insane as to believe that there are individuals on this little obscure planet who are taken into the councils of the Omnipotent Creator—

> "—————— Chosen from above
> "To work exceeding miracles on earth."

Our author accounts for these supposed miracles in the only rational manner, by referring them to the great influence which moral emotions exert over the functions and even the structure of the body.

"But there are certain moral feelings which have a power not only to derange the *functions*, but to destroy the *structure* of certain organs; thus, long protracted grief produces diseases of the liver, heart, and lungs; and the anatomist who examines the body which has sunk under the workings of a wounded spirit, will find the sentiment embodied in the disorganized liver, the tuberculated lungs, or the flaccid and extenuated heart. Again, diseases of physical origin in the heart, liver, or lungs, excite the corresponding moral affections with which these organs are associated; thus, a palpitating heart fills the bosom with vague terrors, and a torpid liver entails all the horrors of hypochondriasis.

> "The yellow bile that on your bosom floats,
> "Engenders all those melancholy thoughts.
>
> "DRYDEN." 13.

If the moral emotions be capable of producing disease, they may also, under certain circumstances, be capable (though more rarely) of effecting cures—especially where there is no organic disease; and no instance of organic disease has been proved in any of the patients. It may be remarked, that it is almost invariably women (who are far more susceptible of moral impressions than men) who have been the "chosen vessels" for enthusiasm, and the most approved subjects for delusion. It was well observed by the sagacious Selden, that "when priests come into a family, they do as a man who would set fire to a house—he does not put his torch to the brick wall; but he thrusts it into the thatch—*so the priests work upon the women, but let the men alone.*"

In the cases of Miss Lalor and Mrs. Stuart, our author could not discover any evidence whatever of change of structure, but merely long continued *nervous affection*—a sufficient explanation of the *suddenness* of the cure. It is difficult to conceive (as our author well observes) a situation better calculated to exalt the imagination, and engross every faculty of the soul, than that of a young enthusiast, who, hopeless of relief from severe suffering, throws herself on the Divine mercy, believing that at *that instant* her case is specially commended to his mercy by one who has already found favour in his sight. She feels that her fate is in suspense—adds prayer to prayer—finds her enthusiasm increase and her corporeal sufferings consequently diminish—imagines the Deity is relenting—and at length sinks to the ground, overwhelmed by the force of her gratitude and her love.* No wonder that such a moral conflict should occasionally work a revolution in the nervous system of a hysterical or hypochondriacal female. And this is the whole secret.

We would add one word of advice to our brethren. They should never, on any account whatever, give oral or written testimonies that can, in the remotest manner, countenance these delusions. We do not, for a moment, suppose that such respectable physicians as Drs. Cheyne and Mills have given improper testimonials—but we do think that it was imprudent to give any testimonials at all. No physician should suffer his name to appear in connexion with superstitions that disgrace the age in which we live. These things should be left to needy, ignorant, and unprincipled adventurers, who, as in Johannah Southcoate's case, caught at the tide of popular credulity, to raise themselves into temporary notice—soon to sink beneath its waves in utter contempt, never to rise again!

The illustrations, which our author appends to his little work, of the influence of the mind over the body, are apposite enough, and might be extended *ad infinitum*. We return him many thanks for his ingenious and entertaining brochure.

---

2. *Functions of the Brain.* M. Flourens' experiments, of which we gave some account in a former number, tend to show that the encephalon is composed of several parts *performing essentially distinct functions.* To explain the reason why certain substances introduced into the vascular or digestive apparatus, while acting on the brain, produce phenomena essentially different, M. Flourens has made numerous experiments. From these experiments he thinks himself authorized to conclude, that each medicinal or other substance produces its proper or specific effect on some particular part of the brain. The experiments themselves not having reached us, we can only give the results. These are:—1st, That, up to a determinate dose, opium acts exclusively on the cerebral lobes; belladonna on the corpora quadrigemina; and alcohol on the cerebellum.

2d, That the physical results of the action of each of these substances on each of these parts are absolutely the same as those from mechanical lesion of these parts. As, for example, when a substance acts only on the lobes of the cerebrum, the functions of those lobes are alone injured: when on the cerebellum solely, those of this organ are deranged; and when on the corpora quadrigemina, those of this organ are injured, &c.

3d, That the action of each substance always leaves, after death, and points out, even during life, signs which may serve to distinguish the affected organ from the others.

* Mrs. Stuart's affidavit.

4th, That camphor, æthers, &c. act in a manner analogous to alcohol; the watery extracts of henbane and bitter lettuce, &c. in a way similar to opium, &c.

The experiments on which the above are founded have been repeated before Cuvier, Humbold, Portal, Dumeril, &c. who were commissioned by the institute to report upon the memoire.—*Revue Med.*

We refer our readers to some remarks which we have made on these kinds of experiments, as performed by M. Magendie, at the end of this department of the Periscope.

---

3. *Loss of Speech and Hearing.* Mr. Thompson, of Whitehaven, has related a curious case of this kind in the April number of the Medical Repository. A female child, in her 18th month, was suddenly seized with convulsions, after which she was observed to have lost her speech and likewise her hearing. But her vivacity remained, and her health was unimpaired. In this state she continued till her sixteenth year, when, after the noise of a public rejoicing, she was observed to have recovered the sense of hearing, and soon afterwards she began to articulate, but made slow progress.

---

4. *Rudiments of a Fœtus in the Abdomen of a Boy.* We have never attached much importance to the marvellous in medicine. They are far more curious than useful. They sometimes illustrate Physiology; but are incapable of contributing to the improvement of any other branch of medical science. Cases of monstrosity, for instance, are more eagerly listened to than the most instructive cases of pathology; and therefore we must sail occasionally with the current of human curiosity, though we shall indulge that passion rather less than some of our neighbours. The fœtus found in the abdomen of a boy, in this country, a few years ago, excited nearly as much attention as Bonaparte's return from Elba, and since that period several other remarkable localities for the germ of man have been discovered. A case was lately read at the Medico-Chirurgical Society of the rudiments of a fœtus in the thorax of a woman who was supposed to labour under disease of the heart. At length an abscess burst, and discharged a misshapen mass having jaw bones, teeth, and other portions of human structure in its involucra. The able author of the paper now before us [M. Breschet] has also transmitted to the same society a drawing of a fœtus found in the substance of the uterus itself, whither it had doubtless made its way from the fallopian tube, in its descent from the ovary to the cavity of the womb.

The case before us happened several years ago, but the particulars have never before been regularly published.

*Case.* Amedée Bissieu was born in the year 1790, during the storms of the revolution, by which his mother was greatly agitated while pregnant. The child was very feeble, but lived. From the time he was able to speak he complained of pain in the left side of his chest and abdomen, which side was more protuberant than the right, giving rise to suspicion, at one time, that there was curvature of the spine. The young Amedée, however, grew up, went to school, and even became an expert rider. He still complained of his side. When in his 14th year he was suddenly seized with acute pain in the left side and left hypochondrium, accompanied by fever. A tumour now appeared in the lower part of the abdomen, for which he was bled and purged. The fever continued, and the tumour made progress. On the 7th day of this attack, M. Blanche, a surgeon, examined him, and ascertained the existence of a tumour the size of a melon in the left side of the abdomen. The youth died in the same year, six months from the invasion of the last attack, worn out, of course, with hectic fever.*

On dissection, a large membranous pouch was found, adhering to all the neighbouring parts, and particularly to the colon, between which and the pouch there was a communication. When the pouch was opened it was found to contain a purulent looking fluid, and two bodies of nearly equal size. One of these consisted of hairs matted together, and formed into a ball the size of a man's fist. The other was an imperfect fœtus, consisting of flesh, bones, &c. covered with skin. From the middle of this mass a short and thick ligament passed to the parietes of the cyst, to which it was attached.

Some doubts having been expressed by the faculty respecting the sex of Bissieu, the corpse was afterwards disinterred and minutely examined, when there was not found any thing to disprove his being of the masculine gender.

A great many pages are taken up with minute descriptions of the monstrosity; but these we do not deem it necessary to dwell upon.—*Archives.*

---

5. *Superfœtation.* As this process is doubted still by many physiologists, the following case, on the authority of Drs. Norton and Stearns, (from the New York Medical Repository,) is deserving of a place in our records of facts.

On the 20th October, 1823, Dr. Norton was called to Mary Johnson, a woman of colour, aged 24 years, and of robust constitution, who had been in labour about two hours before Dr. N's arrival. She was quickly delivered of a *perfectly black child,* one month prematurely, according to the mother's reckoning. The child lived only two hours. On attempting to introduce the hand for the extraction of the placenta, the vagina was found so contracted as to prevent that operation. At the end of six hours Dr. Stearns was called in, in consultation, who advised the exhibition of ergot of rye. This was given in the ordinary doses, and soon produced its usual effects on the uterus. A quantity of water was first discharged, followed by a well-formed fœtus, apparently of four months, and *perfectly white.* The umbilical cord was separated, and the child shewed some signs of life. The placenta came away rea-

---

* Six weeks before the patient's death he passed by stool a small ball of hairs.

dily, and the mother recovered quickly. Both fœtuses are preserved by Dr. Norton, and have been shewn to numerous medical men of New York, who all agreed as to the entire difference of colour—of size—of development of parts—of the epochs of conception (an interval of at least four months)—and finally of fathers—one being a white and the other a negro.

On the above case (which certainly appears to be well authenticated) we shall only observe that there is some little difficulty in explaining the cause why one child should have been *perfectly* white and the other *perfectly* black, admitting that the fathers of them had been so. It is a general law in fœtation that the progeny shall have a hue *intermediate* between the father and mother, when these last are of different colours, as is instanced on a large scale in the mulatto race. In the present case, by a woman of colour is meant, of course, a mulatto woman. Between her and a white man the progeny would be *nearly* white—between her and a negro, the child would be *nearly* black—but not *perfectly* white or black, as is represented by Drs. Norton and Stearns. The difference of hue, however, in a case of superfœtation of this kind, and, under these circumstances, would be so great as to authorize the terms white and black.

---

6. *Functions of the Corpora Striata and Tubercula Quadrigemina.* By M. MAGENDIE.

None can entertain a higher degree of respect for the talents, the zeal, and the "improbus labor" of M. Magendie than ourselves. But we confess that the extent to which vivisections are now carried by that illustrious physiologist and others on the Continent, begins to stagger us a little. Our readers will not accuse us of any affected sensibility or squeamishness respecting experiments on animals, where any important point of practice—or even *physiology* is likely to be elucidated thereby. But when, from mere curiosity, we get into habits of slicing animals to death in the most slow and cruel manner, merely to observe the phenomena presented by the tortured subjects of these experiments, we cannot but recoil, and enquire whether the results of these vivisections are likely to compensate for the quantum of unutterable and unimaginable torture inflicted on animals placed under our dominion by the "Father of All," for our comfort and use, but not for our abuse. We put it to the good sense, the humanity, and the professional knowledge of our brethren, whether *therapeutics* are ever likely to be improved by such experiments as the following—on the *action of medicines.*

"In the course which I gave this winter (says M. Magendie) on the *action of medicines*, I had placed in the ventricles of the brain a grain of emetic tartar, in powder, and by chance the substance was more especially in contact with the corpora striata. After the antimonial had remained there for a quarter of an hour, all the assistants, as well as myself, were not a little surprised to observe the *animals spring forward*

and run *with surprising agility.** I know not how many *conjectures* passed in our minds, without the true one (conjecture) occurring to us; but, on repeating this experiment, and observing that the corpora striata were *nearly destroyed* by the chemical action of the emetic tartar, I recognized the real cause of the phenomena: so that the *integrity* of the corpora striata, in their white portion, is connected with the direction of motion and the *will.*"—*Journal de Phys. Octobre.* 378.

Now to our very plain and humble understandings it does appear somewhat extraordinary that, if the *integrity* of the corpora striata be connected with *motion* and *will,* their almost total *destruction* by tartar emetic should cause the animals to "spring forward and run with surprising agility." If these be the illuminations resulting from slicing the brains of animals—be darkness and a clear conscience our lot!

In fine, we beg to hazard our *conjecture* also—and it is, that the real functions of the different parts of the brain will not be discovered by slicing them down in living animals.

